# Phase I Study of Carbon Ion Radiotherapy and Image-Guided Brachytherapy for Locally Advanced Cervical Cancer

**DOI:** 10.3390/cancers10090338

**Published:** 2018-09-18

**Authors:** Tatsuya Ohno, Shin-ei Noda, Kazutoshi Murata, Yuya Yoshimoto, Noriyuki Okonogi, Ken Ando, Tomoaki Tamaki, Shingo Kato, Takashi Hirakawa, Tatsuya Kanuma, Takashi Minegishi, Takashi Nakano, on behalf of the Working Group for Gynecologic Cancer

**Affiliations:** 1 Gunma University Heavy Ion Medical Center, Gunma University, 3-39-22 Showa, Maebashi, Gunma 371-8511, Japan; noda.saitama.med@gmail.com (S.-e.N.); kazutoshi.m@gunma-u.ac.jp (K.M.); yuya.yoshi@gmail.com (Y.Y.); okonogi.noriyuki@qst.go.jp (N.O.); ken.ando0906@gmail.com (K.A.); tamakit@fmu.ac.jp (T.T.); tnakano@gunma-u.ac.jp (T.N.); 2 Department of Radiation Oncology, Saitama Medical University International Medical Center, 1397-1 Yamane, Hidaka, Saitama 350-1298, Japan; s_kato@saitama-med.ac.jp; 3 Department of Obstetrics and Gynecology, Gunma University Graduate School of Medicine, 3-39-22 Showa, Maebashi, Gunma 371-8511, Japan; thira@gunma-u.ac.jp (T.H.); tminegis@gunma-u.ac.jp (T.M.); 4 Division of Gynecology, Gunma Prefectural Cancer Center, 617-1 Takahayashi-nishi, Ota, Gunma 373-0828, Japan; tkanuma@gunma-cc.jp

**Keywords:** cervical cancer, carbon ion radiotherapy, image-guided brachytherapy, concurrent chemoradiotherapy, phase I study

## Abstract

A phase I study was performed to determine the recommended dose of carbon ion radiotherapy and 3D image-guided brachytherapy for histologically confirmed stage II (≥4 cm), III, or IVA cervical cancer. Dose-limiting toxicities (treatment-related toxicities occurring within three months from the start of carbon ion radiotherapy) included Grade 3 non-hematological toxicity, Grade 4 hematological toxicity, or interruption of treatment for more than two weeks due to treatment-related toxicities. Carbon ion radiotherapy consisted of whole-pelvic irradiation with 36.0 Gy (relative biological effectiveness) in 12 fractions and local boost with 19.2 Gy in four fractions for the primary site, and for positive lymph nodes. Three sessions of three-dimensional (3D) image-guided brachytherapy were administered after completion of carbon ion radiotherapy. Weekly cisplatin at a dose of 40 mg/m^2^ was given concurrently. At a dose level of one, a total rectosigmoid D2cc dose between 67.2 Gy and 71.3 Gy at a biological equivalent dose of 2 Gy per fraction from carbon ion radiotherapy and 3D image-guided brachytherapy was prescribed. Six patients were enrolled into this dose level. No patients developed the pre-defined dose-limiting toxicities. For late toxicities, however, one patient developed Grade 3 rectal hemorrhage requiring transfusion at 10 months after treatment. The median survival time was 50.0 months for the five surviving patients. No further dose escalation was performed, and we determined the dose of level one as the recommended rectosigmoid dose. Although our results are preliminary, the study regimen encourages further investigation (registration: UMIN000013340).

## 1. Introduction

The standard of care for locally advanced cervical cancer is concurrent chemoradiotherapy (CCRT) consisting of external beam radiotherapy (EBRT), brachytherapy, and cisplatin-based chemotherapy. For brachytherapy, the implementation of three-dimensional (3D) treatment planning and dose-volume histogram (DVH) parameter evaluation represents a major advancement in the last decade [1,2]. Recent clinical studies with 3D image-guided brachytherapy (3D-IGBT) have shown increased local control and decreased late morbidities in patients with locally advanced cervical cancer, as compared with historical controls [3,4,5]. On the other hand, large gross tumor volume (GTV) at the time of brachytherapy, large high-risk clinical target volume (CTV_HR_), prolonged overall treatment period, and adenocarcinoma histology were associated with risk of local failure [6,7,8]. Thus, there is a need for improving clinical outcomes by implementing novel dose escalation strategies, improving target dose coverage, shortening the overall treatment period, and incorporating new treatment modalities.

Carbon ion radiotherapy (C-ion RT) offers excellent dose distribution, enabling a concentrated administration of a sufficient dose within a target volume while minimizing the dose in the surrounding normal tissues [9]. Additionally, C-ion RT provides biological advantages not seen in proton or photon therapy, owing to high linear energy transfer (LET); C-ion RT induces increased double-stranded DNA structures, causing irreversible cell damage independently of cell cycle phase or oxygenation, more so than lower LET irradiation such as proton and photon therapy [10].

Several phase I/II clinical studies have been performed to establish an appropriate dose fractionation regimen for C-ion RT with or without cisplatin for locally advanced cervical cancer [11,12,13,14,15,16]. However, a combination of C-ion RT with IGBT has not been evaluated so far. Herein, we report the clinical outcomes of a phase I study (protocol 1202) evaluating C-ion RT, 3D-IGBT, and concurrent weekly cisplatin for locally advanced cervical cancer. The purpose of this study was to determine the recommended dose (RD) for a combination of C-ion RT with 3D-IGBT.

## 2. Materials and Methods

### 2.1. Eligibility

This study was a single-institution phase I study (registration: UMIN000013340), approved by the Institutional Review Boards at the Gunma University Hospital on 22 May 2013 (ethics code: 1041), and conducted in compliance with the Declaration of Helsinki. Written informed consent was obtained from all patients. Inclusion criteria for this study were: (1) histologically confirmed squamous cell carcinoma, adenosquamous carcinoma, or adenocarcinoma of the uterine cervix; (2) International Federation of Gynecology and Obstetrics (FIGO) stage II (≥4 cm), III, or IVA disease without rectal invasion; (3) no para-aortic lymph nodes ≥1 cm in minimum diameter on computed tomography (CT) images; (4) measurable disease; (5) age between 20–75 years; (6) Eastern Cooperative Oncology Group performance status 0 to 2; (7) no prior surgery or chemotherapy for cervical cancer; (8) adequate renal and hepatic function (serum creatinine level, <1.5 mg/dL; bilirubin level, <1.5 mg/mL; and aspartate/alanine aminotransferase level, <100 IU/dL), and bone marrow (hemoglobin level, >9.5 g/dL; leukocyte count, ≥3000/mL; and platelet count, ≥100,000/mL). Patients who had histories of prior chemotherapy or pelvic radiotherapy were excluded from the study. Patients were also excluded if they had severe diabetes mellitus, severe heart disease, severe pelvic infection, severe psychological illness, pregnancy, or active double cancer.

Pretreatment evaluation consisted of an assessment of the gynecological examination, cervical biopsy, routine blood cell counts, chemistry profile, cystoscopy, and sigmoidoscopy. Bladder or rectal involvement was assessed using endoscopy findings. CT scans of the chest, abdomen, and pelvis, and magnetic resonance imaging (MRI) of the pelvis were also performed for all patients. Patients were staged according to the FIGO staging system. Tumor size was evaluated both by gynecological examination and MRI, and the dimensions of the cervical tumor were measured with T2-weighted MRI.

### 2.2. Carbon Ion Radiotherapy

The treatment system of C-ion RT at our facility has been described previously [17]. Carbon ion beams were generated by a synchrotron. The passive scattering technique was applied to the tumor. Beam energies of 290 MeV/u, 380 MeV/u, and 400 MeV/u were employed.

Each patient was positioned in a customized cradle (Moldcare; Alcare, Tokyo, Japan) and immobilized with thermoplastic shells (Shellfitter; Kuraray, Osaka, Japan). Two millimeter thick CT images were taken for treatment planning with a “bladder partially full” status (injection of 100 mL of normal saline) and with a “bladder empty” status (immediately after urination). A glycerin enema for the rectum was performed before obtaining CT images. Three-dimensional treatment planning for C-ion RT was carried out using the XiO-N software (Elekta, Stockholm, Sweden). The radiation dose for C-ions was expressed in Gy (relative biological effectiveness (RBE)), which was defined as the physical doses multiplied by the RBE of the C-ions [18].

**Figure 1:**
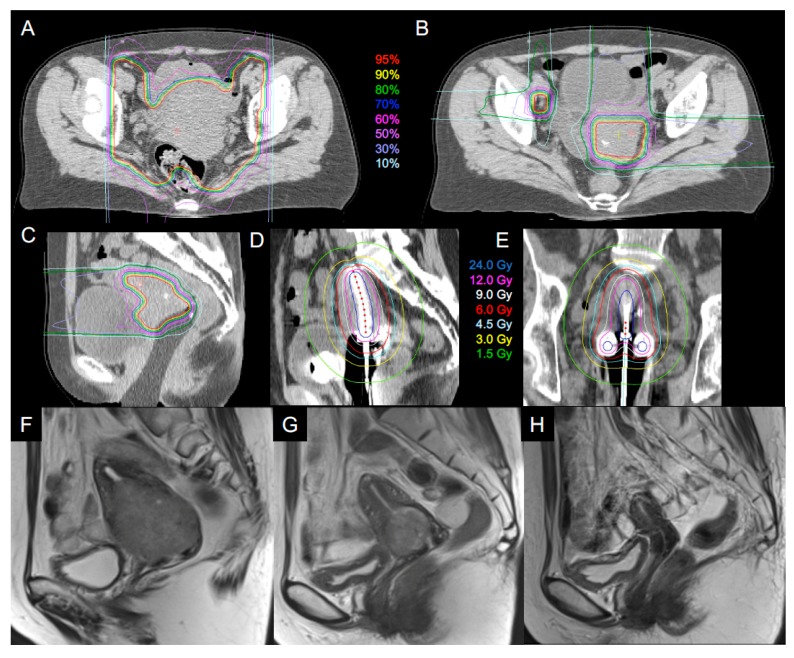
A 57-year-old patient with uterine cervical adenocarcinoma was treated with carbon ion radiotherapy (C-ion RT) and three-dimensional (3D) image-guided brachytherapy (3D-IGBT). This figure shows the dose distribution of C-ion RT for whole-pelvis. (**A**) Boost to primary tumor and positive lymph nodes; (**B**) Dose distribution of C-ion RT boost in sagittal image; (**C**) A vaginal immobilization device was inserted to fix the upper vaginal position and to separate the rectum from the cervical tumor; Dose distribution of 3D-IGBT in a sagittal image (**D**) and a coronal image (**E**); T2-weighted magnetic resonance imaging before treatment (tumor size, 6.4 cm) (**F**); at the end of treatment (**G**); and 36 months after treatment (**H**).

Treatment planning for C-ion RT consisted of whole-pelvic irradiation, and boost irradiation to the primary tumor and to the clinically positive lymph nodes. The gross tumor volume (GTV) was defined by MRI findings and gynecological examination immediately before each treatment planning. The clinical target volume (CTV) of whole-pelvic irradiation included all areas of gross and potentially microscopic disease, which consisted of the primary site (GTV, whole uterus, parametrium, ovaries, and at least the upper half of the vagina) and the whole pelvic node region (enlarged lymph node, common iliac, internal iliac, external iliac, obturator, and presacral node regions). Individual internal margins were also added for each patient based on the uterine body and cervix positions with or without bladder filling (iCTV_WP_). The planning target volume (PTV_WP_) included iCTV_WP_ plus a 5 mm margin for positioning uncertainty. If PTV_WP_ overlapped normal tissues, priority was given to target coverage. PTV_WP_ was encompassed by the 95% isodose line of the prescribed dose. PTV_WP_ was irradiated with the anterior and posterior ports (Figure 1A).

**Figure 2:**
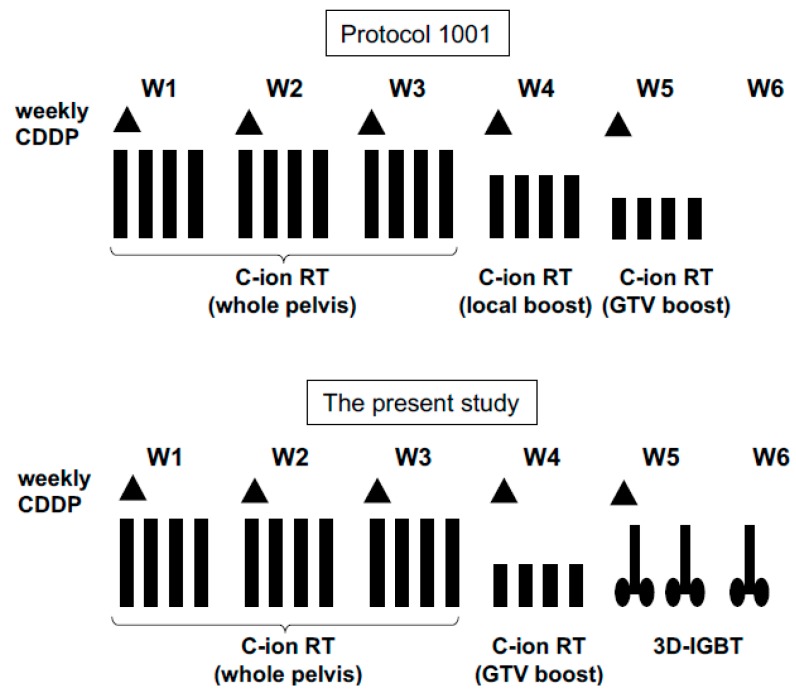
Treatment schema applied for six patients with uterine cervical cancer. Compared with a reference from a previous study (Protocol 1001), the local boost for carbon ion radiotherapy was substituted with 3D image-guided brachytherapy in the present study. CDDP: cisplatin; C-ion RT: carbon ion radiotherapy; GTV: gross tumor volume; 3D-IGBT: 3D image-guided brachytherapy.

After completion of whole-pelvic irradiation, the CTV was reduced to GTV plus cervix (CTV_CX_) and positive lymph node (CTV_LN_). Since the tumor size usually diminished during whole-pelvic irradiation, sequential planning CT images were acquired to define the CTV_CX_ and CTV_LN_ (Figure 1B). For performing this CT, an immobilization device was inserted into the vagina to fix the upper vaginal position and to displace the rectum from the cervical tumor (Figure 1C). The device included three tungsten markers at the cranial side for daily verification of the positioning. Bladder injection of 100 mL of normal saline was performed before acquiring CT images. PTV_CX_ included CTV_CX_ plus a 3 mm margin. However, normal tissue structures such as the rectum, sigmoid, and small bowels in the pelvis plus a 5 mm margin were also excluded from the PTV_CX_ to avoid intestinal irradiation. CTV_LN_ included the residual lymph node, and PTV_LN_ included the CTV_LN_ plus a 3 mm margin excluding the intestines. The dose to PTV_WP_ was fixed at 36.0 Gy (RBE) in 12 fractions over 3 weeks. The dose to PTV_CX_ and PTV_LN_ was fixed at 19.2 Gy (RBE) in 4 fractions over 1 week. Thus, the total dose to the cervical tumor and the positive lymph node was 55.2 Gy (RBE) in 16 fractions over 4 weeks. The C-ion RT dose was prescribed at the isocenter of the PTVs. In the previous study, a standard regimen of C-ion RT without brachytherapy for locally advanced cervical cancer included a total dose to the cervical tumor with 36.0 Gy (RBE) in 12 fractions for whole-pelvis, 19.2 Gy (RBE) in 4 fractions for the primary site, and 19.2 Gy (RBE) in 4 fractions for only the GTV [16]. The dose and fractionation schedule in our study was a modified version of this regimen. The second irradiation phase of the C-ion RT boost to the primary site was substituted with 3 sessions of 3D-IGBT (Figure 2).

Patients received C-ion RT daily for 4 days each week. The patient was positioned on the treatment couch with the immobilization devices at every treatment session, and the patient’s position was verified with a computer-aided online positioning system. Digital orthogonal X-ray images were acquired and transferred to the positioning computer. The positioning images were compared with the reference images which were digitally reconstructed from CT scans. If the difference in positioning set up was >1 mm, the treatment couch was moved until an acceptable position was attained. During whole-pelvic irradiation, patient set up was started immediately after urination to minimize bladder filling prior to irradiation. During the carbon ion beam boost, a bladder injection of 100 mL of normal saline was performed and patient positioning was corrected based on the tungsten markers of the vaginal immobilization device. If necessary, patients were also encouraged to take laxatives to prevent constipation throughout the treatment period.

### 2.3. Image-Guided Brachytherapy

Following C-ion RT, three sessions of high-dose rate brachytherapy were performed twice per week using an Ir-192 Remote Afterloading System (microSelectron, Elekta, Stockholm, Sweden). Our treatment system for 3D-IGBT has previously been described [5]. Tandem and ovoid applicators with or without interstitial needles were used. Three-dimensional treatment planning using in-room CT was performed for each brachytherapy session. The Oncentra Treatment Planning System (Elekta, Stockholm, Sweden) was used for contouring and planning. The CTV_HR_ and organs at risk (OARs) including the rectum, sigmoid colon, and bladder were contoured according to the recommendations for CT-based 3D-IGBT and the Gynecologic Groupe European de Curietherapie, European Society for Therapeutic Radiology and Oncology (GEC-ESTRO) [1,2,19]. To identify the CTV_HR_, findings from gynecological examinations performed at diagnosis and brachytherapy and MRI performed at diagnosis and within 1 week before the first brachytherapy session were used as references. The outer organ contours were delineated to define OARs.

A standard treatment plan was generated based on the Point A-prescribed dose plan using standard source loading patterns and source dwell weights. Optimization of the standard plan was carried out by evaluating source positions and dwell times manually and/or by dragging and dropping isodose lines until the DVH constraints were met with our dose criteria (see the Study Design sub-section). Dose distribution was also evaluated by visual inspection of the isodose lines to avoid excessive heterogeneity (Figure 1D,E). DVH parameters were calculated for each session with respect to the CTV_HR_ D90 and OARs D2cc for the rectosigmoid and bladder.

### 2.4. Cumulative Dose Calculation

The cumulative doses were summarized and normalized to a biological equivalent dose of 2 Gy per fraction (EQD2) using a linear quadratic model with an alpha/beta of 3 Gy for the OARs and 10 Gy for the tumors. In this study, CTV_HR_ D90 and D2cc of the rectosigmoid and bladder were calculated by adding the biologically equivalent doses of C-ion RT and those of all brachytherapy sessions.

### 2.5. Chemotherapy

Five courses of weekly cisplatin at a dose of 40 mg/m^2^ (maximum dose of 70 mg/body) were given during the C-ion RT and brachytherapy period. The first course of cisplatin was administered on day 1 of C-ion RT in principle. Cisplatin was administrated on a different day during the brachytherapy period.

### 2.6. Study Design

The primary endpoint was dose-limiting toxicities (DLT) defined as treatment-related toxicities occurring within 3 months from the start of C-ion RT. DLTs included Grade 3 non-hematological toxicity, Grade 4 hematological toxicity, or interruption of treatment for more than 2 weeks due to the treatment-related toxicities. Secondary endpoints included pelvic control and late toxicities after the start of C-ion RT.

In this study, the C-ion RT dose was fixed but the brachytherapy dose was varied in order to seek the recommended dose for the rectosigmoid. The tolerance dose of the rectosigmoid is usually lower than that of the bladder. Thus, assessment of the rectosigmoid dose level consisting of C-ion RT and 3D-IGBT had first priority over bladder and target doses. The initial prescribed rectosigmoid D2cc dose level was between 67.2 Gy and 71.3 Gy (RBE) EQD2 (Level 1). Six patients were treated at this dose level. If no or one patient developed DLT, the maximum tolerated dose was not determined and this dose level was defined as the recommended dose. If two or more DLTs occurred at the given dose level, the rectosigmoid D2cc dose from C-ion RT and 3D-IGBT was reduced to fall between 63.5 Gy and 67.2 Gy (RBE) EQD2 (Level 0). If this dose level did not cause DLTs, this dose level was defined as the recommended dose.

### 2.7. Evaluation of Safety and Efficacy

The patients were followed-up every week during the treatment period and every month thereafter until 90 days after initiation of C-ion RT. After 90 days, patients were followed-up every 3 months. Acute and late toxicities were classified according to the National Cancer Institute Common Terminology Criteria for Adverse Events, version 4.0. In addition, mucosal reactions of the rectosigmoid colon and sigmoidoscopy findings were evaluated at 1 year after the start of C-ion RT or at the time of rectal bleeding according to the Vienna Rectoscopy Score (VRS) [20]. The worst scores of the rectosigmoid were recorded.

## 3. Results

### 3.1. Patient Characteristics

Six patients were enrolled in this study. All patients completed the planned C-ion RT, IGBT, and chemotherapy. A combination of intracavitary with interstitial brachytherapy was performed in two of six patients (four of 18 sessions). The patient characteristics and DVH parameters are listed in Table 1 and Table 2.

### 3.2. Safety

**Figure 3:**
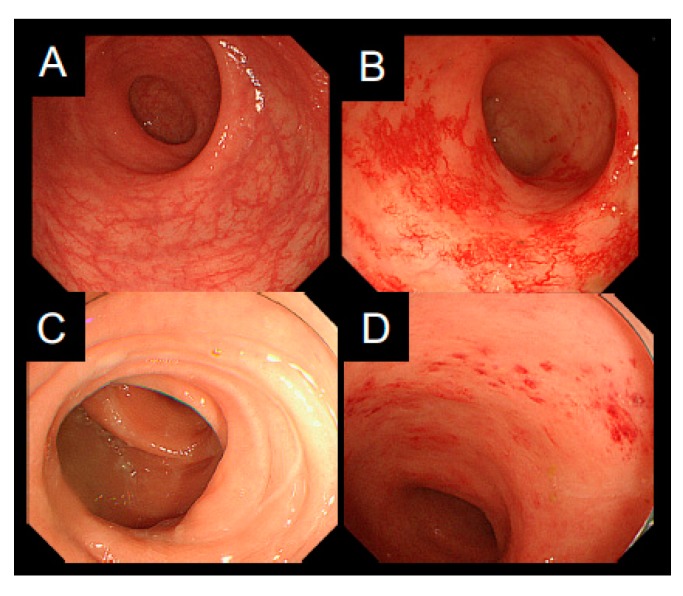
Endoscopic mucosal reaction of the patient (Number 3) who developed Grade 3 late rectal toxicity. (**A**) Before treatment; (**B**) Ten months after carbon ion radiotherapy (C-ion RT) and 3D image-guided brachytherapy (3D-IGBT). The Vienna Rectoscopy Score (VRS) was two. Endoscopic findings of the patient (Number 6) who developed Grade 1 late rectal toxicity; (**C**) Before treatment; (**D**) One year after treatment. The VRS was one.

Acute and late toxicities are listed in Table 3. Grade 2 or higher acute non-hematological toxicities and DLTs were not observed. One patient (Number 3) developed Grade 3 rectal hemorrhage (late toxicity) requiring transfusion 10 months after C-ion RT. Endoscopic findings in this patient showed multiple non-confluent telangiectasia and diffuse, not confluent, reddening of the mucosa, indicating a VRS of two (Figure 3A,B). The estimated total rectosigmoid D2cc from C-ion RT and 3D-IGBT was 68.2 Gy (RBE) EQD2. The patient recovered from the hemorrhage two months after endoscopic ablation and 20 sessions of hyperbaric oxygen therapy. The remaining five patients underwent sigmoidoscopy at one year after the start of C-ion RT. The worst score through the whole rectosigmoid was one in three patients and two in two patients (Figure 3C,D). No patient had a VRS of ≥3.

### 3.3. Efficacy

The clinical outcomes after treatment are summarized in Table 4. The median follow-up time was 47.5 months for all six patients and the median survival time was 50.0 months for the five surviving patients. The first failure site was the lung in two patients, para-aortic lymph node in one patient, and the external iliac node in one patient. The patient with the para-aortic lymph node recurrence also developed recurrence at the uterine body that was outside of both CTV_CX_ and CTV_HR_. Another patient with an external iliac node recurrence received a second C-ion RT with 57.6 Gy (RBE) in 12 fractions and the recurrent tumor has been in control for 30 months. The recurrent external iliac node was recognized as positive before treatment and irradiated with lymph node boost. Based on the retrospective review for dose distribution to the lymph node, the peripheral dose of CTV was reduced to 60% of the planned dose in order to spare intestine dose. This patient had no evidence of disease at the time of last follow-up. Pelvic control was obtained in five of the six patients at the time of last follow-up.

## 4. Discussion

C-ion RT has been in use for more than 20 years in Japan and the efficacy and safety of this therapy, especially for photon-resistant tumors such as bone and soft tissue sarcoma, non-squamous cell carcinomas of the head and neck, pancreatic cancer, and rectal cancer is well accepted [9,10]. For uterine cervical cancer, several dose escalation studies on C-ion RT have been performed without adoption of brachytherapy, mainly due to the lack of 3D dose calculation and evaluation methods for brachytherapy [11,12,13,14,15,16]. Carbon ion beams boost has been used in such studies instead of brachytherapy. However, the high dose boost due to this regimen has frequently resulted in severe late toxicities of the rectosigmoid, including stenosis or perforation in some patients [11,12,15,16]. The current study is the first to adopt brachytherapy in combination with C-ion RT, after the emergence of 3D-IGBT. In our treatment regimen, half of the boost irradiation with carbon ion beams, as in the previous schedule, was substituted with brachytherapy. The pre-defined DLTs were not observed and we determined that the recommended dose for the rectosigmoid was 67.2–71.3 Gy (RBE) EQD2.

In the present study, no patients developed Grade 2 or higher acute non-hematological toxicities of the upper gastrointestinal tract, lower gastrointestinal tract, or the genitourinary tract. Incidence rates of 19%, 10%, and 0%, have been reported for Grade 2 or higher acute non-hematological toxicities of the upper gastrointestinal tract, lower gastrointestinal tract, and the genitourinary tract, respectively, in concurrent C-ion RT with weekly cisplatin [16]. Mundt et al. demonstrated that compared with 3D conformal radiotherapy (3D-CRT), intensity-modulated radiotherapy (IMRT) resulted in fewer patients with Grade ≥ 2 acute gastrointestinal toxicities (60% vs. 91%) and genitourinary toxicities (10% vs. 20%) [21]. Gandhi et al. showed that patients in the IMRT with cisplatin arm experienced significantly fewer Grade ≥2 acute gastrointestinal toxicities (32% vs. 64%) than patients receiving 3D-CRT with cisplatin [22]. Therefore, the incidence of acute non-hematological toxicities in C-ion RT with cisplatin appeared to be lower than that in 3D-CRT. Further comparison of acute toxicities with IMRT will be necessary.

The rectosigmoid has been the critical organ at risk in C-ion RT for cervical cancer [11,12,13,14,15,16]. Therefore, we instituted a careful follow-up of patients post-treatment; endoscopic evaluation was routinely performed at one year according to the VRS. Koom et al. have reported that the probability of a VRS of ≥2 at 12 months was significantly associated with DVH parameters such as D2cc, D1cc, and D0.1cc of the rectosigmoid [23]. Ippolito et al. have demonstrated that a VRS of ≥2 or ≥3 at 12 months correlated significantly with the three-year Grade 2 or higher late rectal bleeding [24]. One patient in our study developed Grade 3 late rectal toxicity at 10 months after the start of C-ion RT, although the toxicity was beyond the pre-defined DLT period. The total rectosigmoid D2cc from C-ion RT and 3D-IGBT in this patient was 68.2 Gy (RBE) EQD2 while the VRS was two. Further accumulation of clinical data will be necessary to understand the relationship between DVH parameters and toxicities or endoscopic score.

In the current study, of six patients, only one developed a recurrent tumor at the uterine body. The combination of C-ion RT and 3D-IGBT may have a potential benefit of increasing central tumor dose while satisfying dose constraints to the rectosigmoid. In previous studies on C-ion RT with or without cisplatin, the two-year local control rates at the recommended dose level were 47% to 71% for adenocarcinoma/adenosquamous carcinoma, and 84% to 100% for bulky squamous cell carcinoma [13,14,15,16]. The major difference between our protocol and that of previous studies was the adoption of IGBT. Theoretically, 3D-IGBT allows accurate high-dose delivery to limited target volumes with rapid dose fall-off gradients. In the target tumor, a highly localized area around the radiation sources receives a much higher dose than that with the use of external carbon ion beams. Furthermore, due to image guidance with applicators, 3D-IGBT enables adaptive and robust dose delivery, especially for a combination of intracavitary and interstitial brachytherapy [25].

In contrast, the potential benefit of C-ion RT as a boost is high LET irradiation and steeper lateral dose fall-off compared with that from brachytherapy. Recently, by using high-resolution microscopy, Oike et al. reported that complex clustered DNA double-stranded breaks were detected in cervical tumor tissue irradiated with C-ion RT but not in tumor tissue treated with X-rays [26]. Nakano et al. compared tumor control between hypoxic and oxygenated cervical tumor groups by assessing oxygen partial pressure [27]. Similar local control rates were recognized between the two tumor groups in C-ion RT, while a significantly worse local control rate in the hypoxic tumor group was found with conventional photon radiotherapy [27]. These translational studies indicated that high LET carbon ion beams may overcome the radiation-resistant nature of tumors by causing severe DNA damage. Most importantly, however, the potential risk of boost with carbon ion beams is inaccurate dose delivery to the target volume caused by anatomical changes such as tumor reduction, and filling of the bladder and rectum during treatment [28]. To minimize this, (1) a vaginal immobilization device was used to displace the rectum, (2) bladder and rectum volumes were carefully controlled within a constant range by bladder injection and with the use of laxatives, and (3) vaginal positioning was reproduced by matching the tungsten markers of the vaginal immobilization device.

C-ion RT has rarely been combined with photon therapy in Japan, whereas German centers have conducted several clinical trials investigating IMRT with C-ion RT boost [29,30]. The optimal combination of C-ion RT with external beam photon therapy or brachytherapy is still controversial. Brahme suggested that the use of quality-modulated radiation therapy (QMRT) with different LET beams shows benefit for tumors consisting of a hypoxic core and the often better-oxygenated microscopic invasive region [31,32]. Based on a biological optimization algorithm, a concomitant boost technique may allow us to deliver a high LET beam to the hypoxic tumor core whereas a low LET photon beam is suitable for the well-oxygenated peripheral region.

## 5. Conclusions

In conclusion, the present phase I study on C-ion RT and 3D-IGBT for locally advanced cervical cancer demonstrated that the pre-defined DLT was not observed and the recommended dose from C-ion RT and 3D-IGBT for the rectosigmoid was 67.2–71.3 Gy (RBE) EQD2. The study regimen was feasible and safe. The major limitation of our study was the small number of patients. However, our results encourage further clinical evaluation of locally advanced cervical cancer treated with C-ion RT combined with 3D-IGBT. We are undergoing a phase II study on bulky squamous cell carcinoma, adenosquamous carcinoma or adenocarcinomas of the cervix in order to evaluate the efficacy and toxicities of this regimen. Also, the present combination of carbon ion RT with brachytherapy may be applied to other gynecological malignancies such as vaginal cancer, endometrial cancer, and post-operative recurrence. In particular, careful evaluation should be performed from the point of view of biological and physical advantage because the optimal combination between C-ion RT and 3D-IGBT is controversial.

## Figures and Tables

**Table 1:** Patient characteristics of six patients with uterine cervical carcinoma enrolled in the study.

Patient Number	1	2	3	4	5	6
Age	45	44	47	56	57	64
Histology	SCC	SCC	Ad	Ad	Ad	Ad
FIGO stage	IIB	IIIB	IIB	IIB	IIB	IIB
Cervical tumor size (cm)	5.7	6	5.7	4.2	6.4	4.8
Pelvic lymph node	Positive	Negative	Negative	Negative	Positive	Positive
Cisplatin (course)	5	5	5	5	5	5
C-ion RT (Gy (RBE)/fr)	55.2/16	55.2/16	55.2/16	55.2/16	55.2/16	55.2/16
Brachytherapy (session)	3	3	3	3	3	3
Overall treatment	34	37	34	35	34	37
time (days)						

C-ion RT: carbon ion radiotherapy, SCC: squamous cell carcinoma, Ad: adenocarcinoma, FIGO: International Federation of Gynecology and Obstetrics, RBE: relative biological effectiveness.

**Table 2:** Dose volume histogram parameters pertaining to therapy regimens for six patients with uterine cervical carcinoma.

Patient Number	1	2	3	4	5	6
Carbon ion radiotherapy EQD2 (Gy (RBE))						
Rectosigmoid D2cc	47.8	44.2	43.2	44.7	45.9	45.6
Bladder D2cc	50.4	73.2	70.9	70.9	71.9	72.0
CTV_HR_ D90	49.7	61.3	61.5	62.7	53.6	45.6
Brachytherapy EQD2 (Gy)						
Rectosigmoid D2cc	22.9	25.7	25.0	23.4	23.7	24.6
Bladder D2cc	29.8	40.1	26.6	40.9	26.3	19.1
CTV_HR_ D90	30.7	28.9	28.6	36.4	30.1	22.3
Total EQD2 (Gy, Gy (RBE))						
Rectosigmoid D2cc	70.7	69.9	68.2	68.1	69.6	70.2
Bladder D2cc	80.2	113.3	97.5	111.8	98.2	91.1
CTV_HR_ D90	80.4	90.2	90.1	99.1	83.7	67.9

EQD: a biological equivalent dose of 2 Gy per fraction, CTV_HR_: high-risk clinical target volume.

**Table 3:** Acute and late toxicities observed in six patients with uterine cervical carcinoma treated with carbon ion radiotherapy and 3D image-guided brachytherapy with cisplatin.

Toxicity	Grade				
	0	1	2	3	4
Acute hematological toxicities					
White blood cell	0	1	4	1	0
Neutrophil	0	5	0	1	0
Hemoglobin	0	6	0	0	0
Platelet	0	1	1	0	0
Acute non-hematological toxicities					
Upper GI tract	3	3	0	0	0
Lower GI tract	3	1	0	0	0
Genitourinary tract	5	1	0	0	0
Skin	4	2	0	0	0
Late toxicities					
Lower GI tract	2	3	0	1	0
Genitourinary tract	5	0	1	0	0

GI: gastrointestinal.

**Table 4:** Clinical outcomes observed in six patients with uterine cervical carcinoma treated with carbon ion radiotherapy and 3D image-guided brachytherapy with cisplatin.

Patient Number	DLT	Disease Status at the Time of Last Follow-Up	OS (months)	PFS (months)	First Failure Site
1	None	DOD	24	3	Paraaortic lymph node
2	None	NED	52	52	
3	None	AWD	50	21	Lung
4	None	AWD	50	7	Lung
5	None	NED	45	45	
6	None	NED	45	15	Left iliac lymph node

DLT: dose-limiting toxicities, OS: overall survival, PFS: progression-free survival, AWD: alive with disease, NED: no evidence of disease, DOD: dead of disease.

## Abbreviations

C-ion RT	carbon ion radiotherapy
3D-IGBT	3D image-guided brachytherapy
DLT	dose-limiting toxicities
RBE	relative biological effectiveness
EQD2	equivalent dose of 2 Gy per fraction
CCRT	concurrent chemoradiotherapy
EBRT	external beam radiotherapy
DVH	dose-volume histogram
CTV_HR_	high-risk clinical target volume
LET	linear energy transfer
RD	recommended dose
FIGO	International Federation of Gynecology and Obstetrics
CT	computed tomography
MRI	magnetic resonance imaging
GTV	gross tumor volume
CTV	clinical target volume
iCTV_WP_	internal clinical target volume with whole pelvis
PTV_WP_	planning target volume with whole pelvis
CTV_CX_	CTV with cervix
CTV_LN_	CTV with positive lymph node
OARs	organs at risk
VRS	Vienna Rectoscopy Score
